# Ablation for atrial fibrillation: CT overlay or standard electroanatomical mapping?

**DOI:** 10.1007/s12471-012-0301-y

**Published:** 2012-07-06

**Authors:** E. E. van der Wall

**Affiliations:** 1 Interuniversity Cardiology Institute of the Netherlands (ICIN)–Netherlands Heart Institute (NHI), Utrecht, the Netherlands; 2 Department of Cardiology, Leiden University Medical Center, Albinusdreef 2, Postal zone: K5-35, P.O. Box 9600, 2300 RC Leiden, the Netherlands

The outcome of catheter ablation procedures for cardiac arrhythmias depends on the ability to evaluate the underlying mechanism and to depict target sites for ablation [1–10]. Image fusion and integration have become routine procedures in the diagnosis and treatment of various cardiovascular disorders [11–16]. Fusion of different imaging modalities within one system may improve electroanatomical modelling and facilitate ablation procedures. Nowadays, three-dimensional (3D) navigation systems are widely used for pulmonary vein antrum isolation [17–24]. However, use of electroanatomical mapping systems may increase procedural duration and overheads, and may limit the choice of ablation catheters. As an alternative to circumvent left atrial mapping, 3D computed tomography (CT) reconstructions of the left atrium can be superimposed directly (CT overlay) on the fluoroscopy image to guide ablation catheters and to mark ablation sites.

In the present issue of *the Netherlands Heart Journal*, Van der Voort et al. [25] describe their initial experience with 3D overlay for ablation of atrial fibrillation. They evaluated the feasibility of the CT overlay technique for pulmonary vein antrum isolation and its equivalence to established electroanatomical mapping. To this purpose, the authors performed circumferential pulmonary vein ablation in 71 patients with atrial fibrillation. They performed 3D reconstructions of the left atrium, which were derived from contrast cardiac CT and circumferential pulmonary vein isolation. CT was initially performed using a 64-slice CT scanner, and later a 256-slice CT scanner, with 85 ml of intravenous contrast. Initial follow-up was scheduled at 2 to 3 months after each ablation, at 6 months, and for every 6 months subsequently. In subsequent ablation procedures, veins were re-isolated, and defragmentation or linear lesions were performed if necessary. Outcome was based on symptoms and subsequent electrocardiographic confirmation of atrial fibrillation or tachycardia. The authors found that adequate 3D reconstructions were formed and registered to fluoroscopy in all patients. All veins, except 2 in one single patient, could be isolated, resulting in freedom of atrial fibrillation in 45 patients (63 %). In 19 patients a second procedure was performed, in which 2.7 ± 1.1 pulmonary veins per patient were re-isolated; in 3 patients a third procedure was performed. After follow-up of 15 ± 8 months, 51 (91 %) patients with paroxysmal and 10 (67 %) with persistent atrial fibrillation were free of arrhythmia.

The authors concluded that the results of 3D overlay for circumferential pulmonary vein isolation are good. The 3D overlay technique is comparable with other techniques, both for paroxysmal and persistent atrial fibrillation. In addition, it was demonstrated that the need for subsequent ablations remains high due to a high incidence of recovery of conduction from the pulmonary veins, but the recovery rate was similar to standard techniques. Therefore, the outcomes of the 3D overlay technique generally appear to be equivalent to other mapping techniques. The authors further claim that, based on other studies, their method of image integration is feasible in terms of handling radiation exposure and having lower costs. However, nowadays both patients and operators are entitled to know the exact radiation dose they receive. Therefore, precise data regarding the total radiation burden should be provided. Along those lines, the potential cost reduction associated with the 3D overlay technique should be given in more accurate terms, certainly when the 3D technique is said to be equivalent to other mapping techniques.

In a previous study by the same group [26], the authors had already successfully evaluated 68 patients with symptomatic atrial fibrillation refractory to medical therapy who were randomly assigned to CT overlay (group 1, *n* = 38) or to a new image integration module called CartoMerge (group 2, *n* = 30). In that study they found that CT overlay for pulmonary vein isolation is feasible and may, in comparison with conventional left atrial navigation systems, shorten procedural time without an increase in radiation burden.

In our centre, Tops et al. [27] had already shown in 16 patients with atrial fibrillation that CT images can be fused with the three-dimensional electroanatomical mapping system in an accurate manner. It was concluded that anatomy-based catheter ablation procedures for atrial arrhythmias may be facilitated by integration of different imaging modalities. Also, Kardos et al. [28] showed that highly accurate CT imaging and the electroanatomical map fusion can be obtained by the Carto 3D electroanatomical mapping system using the coronary sinus as the key anatomical structure for registration. Using this technique the mapping time of the left atrium can be reduced. Finlay et al. [29], however, recently found in the CAVERN trial that CartoMerge appears to be faster and uses less fluoroscopy to achieve registration than 3D image integration using NavX Fusion, but overall procedural times and clinical outcomes were similar.

To summarise, although the current study provides promising data, a prospective, randomised study should be conducted–as the authors also indicated–to adequately compare the current CT overlay technique with standard electroanatomical mapping systems in patients eligible for ablation of atrial fibrillation.
